# The impact of platelets and antiplatelets medications on immune mediation

**DOI:** 10.1016/j.jvssci.2024.100278

**Published:** 2024-12-30

**Authors:** Leela Morena, Isabella Ferlini Cieri, Daniel Marconi Mendes, Sasha P. Suarez Ferreira, Shiv Patel, Samir Ghandour, Maria Fernanda Andrade, Mohit Manchella, Adriana A. Rodriguez, Henry Davies, Shruti Sharma, Anahita Dua

**Affiliations:** a Department of Surgery, Massachusetts General Hospital, Boston, MA; b Department of Surgery, Harvard Medical School, Boston, MA; c Department of Surgery, Universidade Salvador (UNIFACS), Salvador, Brazil; d Department of Surgery, University of Leeds, Leeds, UK; e Department of immunology, Tufts University School of Medicine, Boston, MA

**Keywords:** Antiplatelets, Immunology, Platelets, Immune response

## Abstract

**Objective:**

To investigate the mechanisms through which platelets and antiplatelet therapies modulate the immune response and propose directions for future research in this field, with a particular emphasis on their impact on treatment efficacy and surgical outcomes.

**Methods:**

A comprehensive review of recent studies investigating the role of platelets in immune modulation, specifically highlighting their involvement in pathogen recognition, leukocyte recruitment, and lymphocyte activation. Additionally, the review evaluates the impact of antiplatelet therapies, such as aspirin, P2Y12 inhibitors, and glycoprotein IIb/IIIa inhibitors, on immune responses.

**Results:**

Recent studies have emphasized the critical role of platelets in immune-driven applications, namely, atherosclerosis, cancer, viral infections, and sepsis. These studies also suggest that antiplatelet therapies may alter immune responses. However, the precise mechanisms through which platelets and antiplatelet drugs influence immune responses, as well as their effects on post-treatment and surgical outcomes, are not yet fully elucidated.

**Conclusions:**

Recent studies highlight the important role of platelets in immune processes, such as in atherosclerosis, cancer, viral infections, and sepsis, and suggest that antiplatelet therapies can influence immune responses. However, the exact mechanisms by which platelets and antiplatelet drugs modulate these responses remain unclear. This area presents valuable opportunities for future research to uncover these mechanisms, which could lead to novel therapeutic strategies and better clinical outcomes for patients.

Platelets, a population of small, anucleated cells that circulate in the bloodstream, serve as critical sentinels of vascular integrity. These cells are essential regulators of hemostasis and thrombosis. Dysregulation of these processes is a key factor in the pathophysiology of several diseases associated with high morbidity, such as myocardial infarction (MI), stroke, and venous thromboembolism.^1^ Recent research, however, reveals that platelets extend their functions beyond hemostasis and thrombosis, acting as active inflammatory effector cells. In this capacity, they participate in the orchestration of both innate and adaptive immune responses, highlighting their broader immunomodulatory roles within the body's defense mechanisms. Through functions such as pathogen recognition, regulation of vascular permeability, priming of adaptive immune responses, and recruitment of lymphocytes to sites of peripheral inflammation and infection, platelets likely bridge immune regulation and hemostasis.^2^^,^^3^ This integrative function facilitates a coordinated and effective physiological response, ensuring both vascular integrity and an appropriate immune reaction to pathogenic threats.^2^^,^^3^

Platelets were shown to play roles in both arms of the immune system (Fig). In the innate immune arm, platelets have been shown to express the full spectrum of Toll-like receptors (TLR1 to TLR10), which serve as critical pattern recognition receptors (PRRs). These receptors detect pathogen-associated molecular patterns and damage-associated molecular patterns, enabling platelets to initiate cell-intrinsic or cell-autonomous innate immune responses. Upon activation of these PRRs, platelets are capable of releasing a diverse array of cytokines, chemokines, and other bioactive mediators, engaging essential cellular components of the innate immune system: endothelial cells, neutrophils and monocytes.^4^ Platelets are also responsible for *linking* innate and adaptive immunity by activating dendritic cells (DCs) to subsequently present antigens to T cells.^5^^,^^6^ Furthermore, platelets influence the adaptive immune system by modulating T cell responses through the release of δ-granules, which contain a variety of cytokines and immune mediators.^4^

**Fig:**
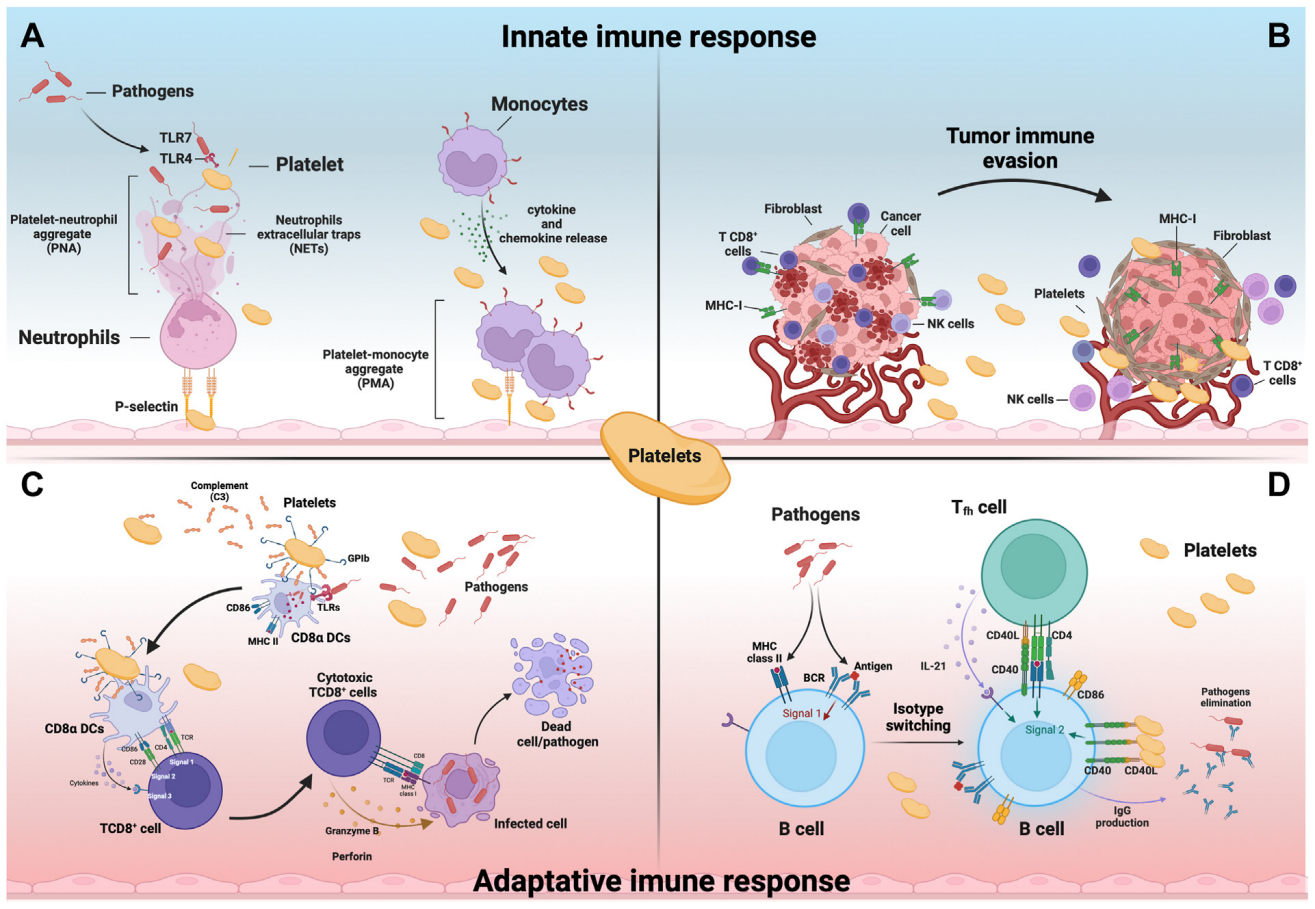
Modulatory mechanisms carried out by activated platelets in the body's innate and adaptive immune responses, showing **(A)** the role of platelets in the formation of platelet-neutrophil aggregate (*PNA*) and in the production of neutrophils extracellular traps (*NETs*) as well as in the formation of platelet-monocyte aggregation (*PMA*) and innate immune cell adhesion on the endothelial wall. **(B)** Tumor evasion promoted by platelets through the stimulation of fibrinogen production by fibroblasts, reducing the exposure of tumor major histocompatibility complex (MHC) I molecules. **(C)** The role of platelets in redirecting pathogenic antigens to CD8α^+^ dendritic cells (DCs) and in promoting cytotoxic CD8^+^ T cell effector mechanisms against cells infected with intracellular pathogens and **(D)** role of platelets in isotype switching and antibody production by B cells activated through CD40-CD40L signaling.

Recognizing the emerging role of platelets in the immune system prompts the question of whether antiplatelet therapy, presently employed for treating cardiovascular disease, impacts post-treatment outcomes by not just regulating thrombotic events, but also modulating the immune environment. Moreover, it also posits the use of antiplatelet therapeutics for potential applications in chronic inflammatory and infectious diseases through their modulation of the immune system. Here we aim to examine and summarize the current literature on antiplatelet therapy and its capacity for immune modulation.

## Methods

This comprehensive review investigates the relationship between platelet aggregation inhibitors and immune system phenomena. A search was conducted across databases like PubMed and Medline, using the keywords: platelet aggregation inhibitors" or "antiplatelet drugs" or "antiplatelet agents" or "platelet aggregation inhibitors" or "antiplatelet therapy" and "immune system" or "immune response" or "immune function" or "immune regulation" or "immunomodulation" or "immunophenomena.” The search was made in November 2023. Studies unrelated to this intersection of key words, non-English language articles, study case reports, editorials, commentaries, and non-peer-reviewed literature were excluded. The papers were screened initially based on their titles and abstracts and for a final selection, a full-text review was made. Additionally, reference lists of included papers were hand searched to identify additional relevant studies that might have been missed during the initial database search. This review did not require institutional review board approval, as it did not involve the collection of data from human participants. BioRender was used to create the Figure.

## Platelet activation and thrombosis

Platelets are integral components of the circulatory system performing a range of essential functions crucial to maintaining vascular homeostasis and immune defense. These functions include (a) the formation of a provisional clot at the site of the vascular injury, (b) the secretion of bioactive molecules, (c) the induction of vasoconstriction to mitigate hemorrhage, (d) the activation of the coagulation cascades, (e) facilitation of tissue repair and regeneration, and (f) the orchestration of immune-hemostatic responses.^7^ These processes are initiated by the adhesion of platelets to sites of vascular injury, facilitated by a reduction in their velocity within the bloodstream. This deceleration enables platelets to bind to exposed collagen on the damaged endothelial surface, primarily through interactions between platelet glycoprotein (GP) Ia/GPIIa integrins and collagen fibers. This initial binding promotes further platelet-collagen interactions via the GP6 receptor, which triggers transmembrane and intracellular signaling cascades within the platelets.^6^ After adhesion to the injured vessel wall, critical activation pathways are triggered within the platelets, resulting in their aggregation.^8^ Upon activation, platelets discharge the contents of their storage granules—α-granules, δ-granules, and lysosomal granules, each laden with a diverse array of bioactive molecules, including coagulation factors, adhesion molecules, growth factors, and angiogenesis regulators.^2^ In addition, platelet granules contain a diverse repertoire of proinflammatory and anti-inflammatory cytokines and chemokines, which were previously thought to be unrelated to clotting processes. For example, transforming growth factor-beta, a cytokine with dual roles in promoting and inhibiting inflammation, is abundantly present in circulating platelets.

However, its precise function in platelet-mediated immune responses remains to be elucidated fully, underscoring the complexity of platelet involvement in both immune regulation and hemostasis.^2^ Upon activation, adherent platelets undergo a striking morphological transformation, transitioning into an irregularly spherical shape with numerous filopodia extending toward the subendothelium.^3^^,^^6^ This structural change enhances platelet proximity, promoting increased platelet-platelet interactions and facilitating the formation of aggregates. Concurrently, the release of biochemical agents from platelet granules, including adenosine diphosphate (ADP), thromboxane A2 (TXA2), and serotonin, initiates a positive feedback loop that recruits and activates additional platelets, amplifying the aggregation process in a cascading manner.^6^^,^^9^ These agents are recognized by specific receptors on the platelet membrane, most notably GP2b/GP3a, thus triggering an intracellular signaling pathway further amplified by TXA2 and serotonin, ultimately potentiating vasoconstriction and resulting in a physiological vascular spasm/constriction that prevents blood loss at the injury site.^10^

The coagulation cascade, fundamental to hemostasis, involves two primary pathways: intrinsic and extrinsic.^11^ The intrinsic pathway is initiated when factor XII is activated upon contact with negatively charged surfaces, such as activated platelets. This activation sequentially triggers factors XI and IX, ultimately resulting in the activation of factor X. In contrast, the extrinsic pathway is activated by tissue factor (factor III) released from damaged tissues. In the presence of calcium ions and platelet factor 4, tissue factor activates factor VII, converting it to factor VIIa, which subsequently activates factor IXa or directly activates factor Xa. Both pathways converge at the activation of factor X, which is a crucial step in the coagulation cascade, leading to thrombin generation and fibrin clot formation.^7^ Thrombin catalyzes the conversion of fibrinogen into fibrin, which polymerizes to form a stabilizing meshwork essential for clot integrity.^12^ Platelet activation and contractile proteins such as actin and myosin will then facilitate platelet adherence to the vessel wall via the fibrin network. Fibrinolysis, the process responsible for clot degradation, is primarily mediated by tissue plasminogen activator, which converts plasminogen to plasmin, the enzyme responsible for fibrin disassembly. Antifibrinolytic agents like tranexamic acid play a pivotal role in reducing clot degradation and stabilizing clots by inhibiting plasminogen activators, thereby ensuring precise regulation of clot formation and resolution.^13^ Additionally, to aid vascular hemostasis, platelets secrete numerous cytokines, chemokines, and cell surface receptors, which activates and recruits leukocytes to the platelet adhesion site in the blood vessel, which is also known as the platelet-mediated immune response.^3^

## The interplay between platelets and the immune system: an exploration of current knowledge

### Platelets and the innate immune system

Platelets influence both thrombotic and immune processes, in part, through their interactions with endothelial cells. A central mediator of this effect is CD154 (also known as CD40L), a protein commonly associated with CD4^+^ T cells, but found in substantial quantities within platelets. Upon platelet activation, CD154 is rapidly expressed and released, playing a pivotal role in modulating endothelial cell function. This interaction not only enhances platelet adhesion and aggregation but also triggers proinflammatory and prothrombotic responses within the endothelium, underscoring the multifaceted role of platelets in both vascular and immune regulation.^14^^,^^15^ The impact of platelet activation on endothelial cells is evident in the upregulation of key endothelial adhesion molecules, such as E-selectin, vascular cell adhesion molecule-1, and intercellular adhesion molecule-1.^2^^,^^16^ These molecules facilitate leukocyte adhesion and transmigration across the endothelium. Additionally, platelet activation induces the endothelial release of chemokines, including CC-chemokine ligand 2, and cytokines such as interleukin-6, both of which are crucial for the recruitment and activation of leukocytes at sites of inflammation.^2^^,^^16^ This coordinated response illustrates the role of platelets in linking hemostatic processes with immune surveillance and inflammation.^2^^,^^16^

Aggregation also creates a localized environment that facilitates intracellular signaling cascades, enhancing the recruitment and activation of immune cells at sites of vascular injury or inflammation. For example, activated platelets can synthesize interleukin 1β, a potent proinflammatory cytokine, highlighting a direct role for platelet aggregation in immune response modulation.^17^ The extent of platelet aggregation and the overall platelet count are likely to modulate the magnitude of immune responses. Aggregated platelets release cytokines and other soluble mediators, which amplify paracrine signaling and immune cell activation. This phenomenon has been observed in innate immune contexts, where platelet aggregation and surface molecule expression are critical for neutrophil and monocyte activation.^18^ However, it remains uncertain whether platelet aggregation is an absolute requirement for immune activation and ligand expression or whether individual, nonaggregated platelets can exert similar effects.

As previously discussed, platelets express a variety of types of PRRs, including TLRs 1-10 and Nod-like receptors. This repertoire of PRRs enables platelets to detect both intracellular and extracellular damage-associated molecular patterns and pathogen-associated molecular patterns, influencing their role in coordinating the innate immune system.^10^, ^11^, ^12^, ^13^, ^14^ Studies by Koupenova et al demonstrate that, in both human and mouse platelets, the activation of TLR7, a PRR that senses single-stranded RNA, induces significant morphological changes in platelets. This activation promotes interactions with neutrophils, resulting in the formation of platelet-neutrophil aggregates (PNAs). Moreover, the study reports that platelet-deficient mice exhibit a higher mortality rate when infected with the positive-strand RNA virus, encephalomyocarditis virus, underscoring the critical role of platelets in antiviral immune responses and host defense against viral pathogens. Overall, the transfer of TLR7-expressing platelets into TLR7-deficient animals promoted a significant increase in the formation of PNAs, along with a reduction in mortality in encephalomyocarditis virus-infected mice.^19^

In another study, TLR4, a receptor that detects the bacterial cell wall component lipopolysaccharide (LPS), was identified as a key modulator of platelet-neutrophil interactions. Clark et al^20^ demonstrated that human platelets could bind to LPS in a TLR4-dependent manner by treating platelets in vitro with fluorescently labeled LPS. When platelets were treated with LPS in conjunction with eritoran, a TLR4 antagonist, the binding of platelets to LPS was significantly reduced, confirming the role of TLR4 in this process. Furthermore, platelets treated with LPS or plasma from septic patients exhibited increased neutrophil adhesion and an enhanced formation of neutrophil extracellular traps in vitro. The study also showed that, in vivo, mice infected with *Escherichia coli* displayed significant bacterial trapping within PNAs in liver sinusoids. Depletion of either platelets or neutrophils in these mice led to a marked reduction in bacterial trapping, underscoring the essential role of PNAs in the immune defense against bacterial infections.^20^

Another example of platelet-leukocyte interaction in innate immunity is the formation of platelet-monocyte aggregates (PMAs), which occurs as a result of P-selectin expression on the surface of activated platelets.^21^ The formation of PMAs has already been linked to poor outcomes in several diseases.^22^^,^^23^ In a prospective study, elevated PMA levels were linked to a higher mortality risk in older patients with sepsis, even after adjusting for confounding variables. PMA levels greater than or equal to 8.43% during sepsis were the best predictor of 28-day mortality in older patients, with an area under the receiver operating curve of 0.82.^23^ PMA levels were also found to be an early marker of acute MI (AMI). A study performed with 211 patients admitted to the emergency department with chest pain showed that patients with AMI had significantly higher PMA levels compared with those without AMI.^22^ Similarly, a study by Dev et al^24^ demonstrated that patients with cryptogenic stroke (CS) and cardioembolic stroke (CE) exhibit significantly elevated levels of PMA and PNAs compared with healthy controls. Additionally, platelets from CS and CE patients showed increased expression of P-selectin and significantly elevated intracellular calcium levels, in contrast with platelets from healthy individuals. These changes were associated with the hypercoagulable state observed in the blood of both CS and CE patients. The study shows the critical role of platelet interactions with innate immune cells in the pathophysiology of conditions such as sepsis, AMI, and stroke (both cryptogenic and cardioembolic), highlighting the need for a deeper understanding of these cellular interactions to better inform therapeutic strategies.^24^ For instance, while the heightened proinflammatory activity of these innate immune cells can be partially explained by the formation of PNAs and PMAs, which promote direct cell-cell interactions, additional mediating signals from platelet granules—such as the release of cytokines and chemokines—are likely to contribute significantly to this phenomenon. This further underscores the importance of elucidating the mechanisms underlying platelet-mediated modulation of neutrophil and monocyte effector functions, because these processes may play a pivotal role in the development and progression of conditions like sepsis, AMI, and stroke.

In addition to neutrophils and monocytes, natural killer (NK) cells can also be affected by platelets. NK cells are prototypical innate immune cells, critical in early recognition and elimination of cancer and infected cells, which they kill by using cell-surface receptors that detect the lack major histocompatibility complex class I molecules (MHC-I) on transformed and/or infected cells. In the context of cancer, the interaction between NK cells and platelets significantly alters NK cell function during anticancer immune responses.^25^, ^26^, ^27^, ^28^, ^29^ A study by Nieswandt et al^25^ demonstrated that platelets can aggregate around tumor cells and protect them from NK cell-mediated antitumor responses in vitro; consistent with this finding, the injection of CFS1 (fibrosarcoma), ESb (lymphoma), or B16F10 (melanoma) cells into platelet-deficient B6 mice significantly reduced the metastatic capacity of these tumor cells in vivo, highlighting the crucial role of platelets in facilitating tumor cell immune evasion and metastasis.^25^ In another study, the injection of Lewis lung carcinoma cells into wild-type (WT), *Gαq*^*−/−*^ (with defective platelet activation) or *Fib*^*−/−*^ (with fibrinogen deficiency) resulted in a significant decrease in survival and metastasis of tumor cells injected in the *Gαq*^*−/−*^ and *Fib*^*−/−*^ animals when compared with WT controls, indicating that immune evasion promoted by platelets arises from their activation and ability to coat cancer cells with fibrinogen, which probably shields them from NK cell recognition and elimination, thereby enhancing cancer cell survival and metastatic potential via evasion of the NK-mediated immune response.^26^ Another mechanism by which platelets were shown to assist cancer cells evasion from NK cells antitumor responses is through the transfer of their own MHC-I molecules to tumor cells. By incubating three distinct tumor cells lines that constitutively express no, low, and high MHC I levels (NCCIT, SKBR-3, and PC3, respectively) with platelets, Placke et al^27^ demonstrated that tumor cells incubated with platelets showed expression of CD61 (a platelet marker) and high-levels of platelet-derived MHC-I on their plasma membrane. Additionally, they have shown that platelet-coated tumor cells significantly reduce NK cell cytotoxicity and interferon-γ production. And blocking MHC-I on these tumor cells partially restores both functions, highlighting the platelet-derived MHC-I's role in this immune evasion mechanism of tumor cells because such transfer of MHC-I molecules enables cancer cells to compensate for the reduced or lack of expression of their own MHC-I, ultimately evading detection by NK cells and T cells.^27^^,^^28^

Although these studies demonstrate that platelet-induced immune evasion can impair NK cells' antitumor responses, no research has yet assessed how platelets might affect NK cells' crucial role against intracellular pathogens. Therefore, given the role of platelets in tumor cell evasion from the immune system, it would be interesting to investigate whether similar mechanisms could also be involved with the evasion of virus- or intracellular bacteria-infected cells from NK cell-mediated responses, thus providing valuable insights into the broader impact of platelets on immune modulation.

Platelets have also been linked to other cells within the innate immune system. Eosinophil activation by platelets has shown to occur during type II immune responses in diseases like asthma.^30^, ^31^, ^32^ The activation of eosinophil β1-integrin through P-selectin takes place in asthma, leading to the swift migration of platelet-eosinophil complexes into the lungs, particularly in more severe cases.^31^

In addition to their roles in hemostasis and inflammation, platelets also serve as reservoirs for protein glycosylation donor substrates, such as uridine diphosphate sugars. At sites of inflammation, platelets release these uridine diphosphate sugars, facilitating glycosylation modifications of molecules like immunoglobulin G (IgG), thereby modulating immune responses.^33^^,^^34^ Platelets contain and release both glycosyltransferases and sugar nucleotide donors upon activation, providing the necessary components for extracellular glycosylation reactions.^33^ This platelet-mediated glycosylation machinery offers a pathway for modulating immune responses and potentially altering cell behaviors in various disease contexts.^34^ The ability of platelets to supply both enzymes and substrates for glycosylation highlights their importance in dynamic regulation of glycan structures on cell surfaces, which can significantly impact immune cell function and signaling.^33^^,^^34^

### Platelets and the adaptive immune system

Platelets have also been shown to play a role in the adaptive immune response. At sites of infection, platelets may facilitate the efficient activation of naïve T cells by enhancing the function of antigen-presenting cells (APCs). Through their interactions with APCs, platelets can contribute to the priming of T cells, thereby linking innate and adaptive immune responses and further underscoring their immunomodulatory capabilities.^5^^,^^6^ Previous studies have indicated that the intracellular bacterium *Listeria monocytogenes* establishes a rapid association with platelets in the bloodstream, a process that relies on the presence of GPIb and complement component C3. This interaction is critical as it redirects a subset of the bacteria toward splenic CD8α^+^ DCs, professional APCs capable of activating naïve T cells most effectively. These findings suggest that the targeting of *L. monocytogenes* to CD8α^+^ DCs, mediated by a complement-platelet mechanism, plays a pivotal role in the subsequent activation of T-cell immunity.^5^^,^^6^ In the process of T-cell migration, platelets are instrumental in facilitating the adherence of T cells to high endothelial venules in lymph nodes, a preliminary step in the initiation of antigen-specific immune responses.^35^ This process takes place after the binding of platelets to circulating lymphocytes and has demonstrated a role in the CD8^+^ T-cell responses during viral infections by facilitating the migration of T cells into secondary lymphoid organs.^36^^,^^37^ T-cell functions are also regulated by platelet-derived CD154 (CD40L) in vitro and in vivo.^38^^,^^39^

In a study involving CD154-deficient mice immunized with ovalbumin (OVA), the transfer of inactivated platelets from WT mice led to a significant increase in the frequency of OVA-specific CD8^+^ interferon-γ^+^ T cells in the blood. These T cells also exhibited enhanced cytolytic activity when incubated with OVA-expressing target cells in vitro. Because these WT platelets were the only source of CD40L (CD154) in the deficient mice, the findings suggest that platelet-derived CD40L is sufficient to directly modulate T-cell responses in vivo, highlighting the critical role of platelet-CD40L interactions in shaping adaptive immunity.^38^^,^^39^ This same study showed that the transfer of WT inactivated platelets into CD154-deficient mice infected with adenovirus was able to affect B cells responses by stimulating the isotype switch from IgM to IgG, thereby boosting antiviral neutralizing antibody responses in those animals.^39^ Another study investigating the interaction between B cells and platelets demonstrated that, following in vitro coculture, both CD40 and CD40L, expression was significantly upregulated in platelets and B cells. This increase was accompanied by a higher production of IgGs in those B cells, alongside a higher expression of platelet and B cells activation markers (P-selectin and CD86, respectively).^40^

The results from the aforementioned studies illustrate the various mechanisms by which platelets can influence key effector functions of the adaptive immune response. However, several questions remain unresolved. Although platelets have been shown to direct intracellular bacterial pathogens toward professional APCs during infections, it remains unclear whether this mechanism similarly applies to viral infections or chronic diseases, presenting an intriguing avenue for further research with potential to enhance patient care in these contexts. Additionally, platelets' role in the early stages of antigen-specific T- and B-cell responses—facilitating their adhesion to high endothelial venules in lymph nodes—and their modulation of T- and B-cell effector functions through CD40L signaling further establish platelets as significant contributors to the adaptive immune response. By not only recruiting and activating adaptive immune cells, but also modulating the overall effectiveness of antigen-specific responses, platelets emerge as pivotal players in immunity. Given their critical role in the initiation of T- and B-cell responses, future research should explore how platelets may influence the generation and function of memory T and B cells. Such investigations could provide valuable insights into the formation of immunological memory and potentially enhance vaccine efficacy and the treatment of chronic or autoimmune diseases.

## Deciphering the immune modulatory functions of antiplatelets agents

Acetylsalicylic acid, commonly referred to as aspirin, is a cyclo-oxygenase 1 and 2 inhibitor that has been used traditionally for its analgesic, antipyretic, and anti-inflammatory effects.^41^ However, it was not until 70 years after its initial use that its antithrombotic attributes were acknowledged.^41^ The primary mechanism through which aspirin exerts its antithrombotic action is by blocking the synthesis of TXA2 in platelets, which results in the suppression of platelet aggregation.^41^^,^^42^

A cross-sectional investigation was conducted involving 114 patients with type 2 diabetes mellitus, assessing plasma levels of CD40L and urinary concentrations 11-dehydro-thromboxane B2—a stable marker for TXA2 production—after 100 mg/day aspirin treatment for 1 to 6 weeks.^43^ The study observed a marked decrease in CD40L and a consequent decrease in 11-dehydro-thromboxane B levels after treatment.^43^ Additionally, a randomized, parallel trial with varying aspirin doses (30, 100, or 325 mg/day) for 1 week plus a 10-day washout was conducted on 18 patients with type 2 diabetes mellitus.^43^ Results indicated a 40% to 50% decrease in plasma CD40L levels across all doses, persisting at 2 hours, 24 hours, and 7 days, demonstrating the TXA2's role in CD40L release by platelets.^43^ However, the study failed to demonstrate a dose-dependent effect of aspirin on CD40L release, suggesting aspirin's limited modulatory effect on CD40L in diabetic patients.^43^ In another study, platelet-rich plasma from patients treated with 325 mg of aspirin daily for a week displayed a 50% decrease in CD40L levels after collagen-induced activation compared with the platelet-rich plasma before aspirin administration.^44^ Similar findings were described on plasmatic CD40L levels after administration of P2Y12 inhibitor Clopidogrel.^45^ However, aspirin administration showed a lower inhibition of platelet-leucocyte aggregation when compared with P2Y12 inhibitors.^46^

P2Y12 inhibitors, including clopidogrel, prasugrel, and ticagrelor, function as antiplatelet agents by blocking the ADP receptor on platelets. This inhibition decreases the surface expression of GPIIb/GPIIIa receptors, thereby decreasing platelet aggregation. These inhibitors are commonly used in combination with aspirin in a treatment approach known as dual antiplatelet therapy, which is designed to further mitigate the risk of atherothrombotic events, particularly in patients with cardiovascular disease or those undergoing stent placement.^43^

Nevertheless, different studies have demonstrated that the use of such drugs is associated with the inhibition of both PMA and PNA formation.^43^^,^^44^^,^^46^^,^^47^ In fact, the impact of aspirin and P2Y12 inhibitors administration on the formation of platelet-leukocyte aggregates has been explored across various diseases. In the setting of cancer, for example, both medications have been associated with decreased metastases in murine models and in humans.^48^, ^49^, ^50^ In the context of viral diseases, several studies have demonstrated that aspirin and P2Y12 inhibitors suppress the infiltration of cytotoxic T cells and proinflammatory leukocytes into hepatic tissues. As a result, prolonged treatment with these agents has been shown to not only reduce hepatic injury during viral infections, but also to prevent the development of hepatocellular carcinoma in murine models of chronic hepatitis B infection. However, this reduced entry of cytotoxic T cells and proinflammatory leukocytes into the liver is associated with a diminished capacity for viral clearance, suggesting a potential trade-off in the long-term use of aspirin and P2Y12 inhibitors in managing chronic viral infections. This dual effect underscores the need for further investigation into the optimal balance between preventing tissue damage and maintaining effective antiviral immunity.^47^^,^^51^^,^^52^ Aspirin has been linked to a decrease in platelet and immune activation in patients infected with HIV-1 who are already receiving antiretroviral therapy.^53^^,^^54^ Low-dose aspirin has also been associated with a decrease in activated CD4^+^ T cells in cervical samples, which may contribute to increased immunodeficiency in HIV-infected patients.^55^ However, a randomized controlled trial involving 121 HIV-infected patients did not demonstrate any difference in immune activation after 12 weeks of daily aspirin treatment.^56^ Antiplatelet agents have also been identified as potential therapeutics impacting sepsis treatment, because clinical data have shown a correlation between the use of aspirin and P2Y12 inhibitors and lower mortality in patients with systemic inflammatory response syndrome and sepsis.^57^, ^58^, ^59^, ^60^

The GPIIb/IIIa complex constitutes the most prevalent receptor found on the surface of platelets.^61^ Upon platelet activation, triggered by factors such as thrombin, collagen, or ADP, GPIIb/IIIa undergoes a conformational alteration, shifting from a low-affinity to a high-affinity state for fibrinogen, facilitating platelet aggregation.^61^ Additionally, GPIIb/IIIa can bind to von Willebrand factor present on the exposed subendothelial matrix at sites of vascular injury. This interaction facilitates platelet spreading and clot retraction.^61^

The group of GPIIb/IIIa inhibitors include Abciximab (Fab region fragments of monoclonal antibodies against GPIIb/GPIIIa receptors), eptifibatide and tirofiban.^62^, ^63^, ^64^ These medications are administered intravenously and are primarily used in the treatment of non-ST elevation MI and during percutaneous coronary intervention procedures for the management of acute ischemic complications.^62^, ^63^, ^64^ The role of GPIIb/IIIa inhibitors has also been researched as an immune system modulator. In a murine sepsis model, eptifibatide was shown to decrease platelet granzyme B-mediated apoptosis.^65^ Furthermore, the administration of GPIIb/IIIa receptor antagonists in baboons and rabbits resulted in decreased damage and lethality in endotoxin-induced ischemic organ models.^66^^,^^67^ However, suboptimal doses of GPIIb/IIIa inhibitors showed a proinflammatory effect.^38^ In a human study, evaluation of specific cellular adhesion molecules (CD11b, CD54, and CD162) on monocytes revealed no significant effect after the administration of eptifibatide or tirofiban.^68^ Nevertheless, additional human studies are required to gain a deeper understanding of the impact of GPIIb/GPIIIa inhibitors on the immune system.

Indeed, the use of antiplatelet drugs—such as aspirin, P2Y12 inhibitors, and GPIIb/IIIa antagonists—has been studied extensively in contexts such as viral infections, sepsis, diabetes, cancer, and cardiovascular diseases, and further research is needed to explore their impact on other immunological conditions, including allergies, autoimmune disorders, and chronic inflammatory diseases. Such studies could significantly advance our understanding of the role of platelets in various pathological states and lead to the development of more effective therapeutic strategies, enhancing patient outcomes through the optimized use of antiplatelet agents in these conditions (Table).

**Table:** Summary of publications summarizing literature on antiplatelet therapy and its impact on immune modulation

Authors	Context/model	Organism	Antiplatelet drug	Findings/conclusion
Koupenova et al, 2018	Viral infection	Mouse	-	TLR7-induced PNAs enhance viral clearance.
Elzey et al, 2005	OVA-specific T-cell immune response	Mouse	-	Platelet CD40L enhance antigen-specific T cell responses in vivo.
Clark et al, 2018	Sepsis	Mouse	-	Platelet TLR4 promotes NET formation and bacterial trapping.
Verschoor et al, 2011	Bacterial infection	Mouse	Platelet GPIb and complement C3	Platelets redirect bacteria to CD8^+^ DCs and enhance T CD8^+^ immune activation.
Nieswandt et al, 2005	Cancer	Mouse	Platelet aggregation and fibrinogen transfer	Platelets promote tumor cells immune-evasion via fibrinogen coating and MHC I transfer.
Santilli et al, 2006	Type 2 diabetes	Human	Aspirin	Reduction in platelet-derived inflammation and CD40L and TXA2 levels in plasma.
O'Brien et al, 2013	HIV infection	Human	Aspirin	Reduction of platelet and immune activation in antiretroviral-treated patients.
Schrottmaier et al, 2015	PNA aggregates	Human	P2Y12 inhibitors (clopidogrel, ticagrelor)	Reduction of platelet-leukocyte aggregates and P-selectin expression, downmodulating innate immune activation.
Akinosoglou et al, 2014	Sepsis	Mouse and human	Aspirin and P2Y12 inhibitors	Reduced immune activation and improved survival in sepsis models
Gareau et al, 2018	Cancer	Mouse	Ticagrelor	Inhibited platelet-tumor interactions and reduced metastasis.

*DC,* Dendritic cell; *MHC,* major histocompatibility complex; *PNA,* platelet-neutrophil aggregate; *TLR,* Toll-like receptor.

## Conclusions

Platelets, once considered solely as key players in thrombosis and hemostasis, are now recognized for their substantial role in immune regulation. Over the past decade, a growing body of research has demonstrated that platelets actively contribute to both innate and adaptive immune responses through their interactions with immune cells, release of cytokines, and involvement in inflammatory processes. These findings have prompted investigations into whether medications that modulate platelet function, such as antiplatelet therapies, might also have broader immunomodulatory effects. Although the potential for these therapies to influence immune responses in various diseases is intriguing, the clinical application of these insights remains unclear.

The clinical relevance of this study hinges on the ability to translate the immune-modulating properties of antiplatelet drugs into therapeutic strategies for a range of diseases beyond thrombosis, such as chronic inflammatory conditions, autoimmune disorders, and infections. However, current evidence is fragmented and, in some cases, conflicting. Although some studies suggest that antiplatelet therapies, such as aspirin, P2Y12 inhibitors, and GPIIb/IIIa antagonists, can reduce immune-mediated damage, the impact of these drugs on immune regulation, specifically in terms of reducing inflammatory responses or enhancing viral or bacterial clearance, remains poorly understood.

## Limitations

Future research should focus on several key areas. First, elucidating the molecular pathways by which antiplatelet drugs influence immune function, particularly in relation to platelet interactions with leukocytes, APCs, and endothelial cells, will be essential. Understanding these pathways could uncover new therapeutic targets for managing diseases characterized by chronic inflammation or immune dysregulation. Second, clinical studies should aim to identify biomarkers that predict patient responses to antiplatelet therapies in the context of immune modulation. Such biomarkers could help stratify patients and tailor therapies to those most likely to benefit from immune regulation without compromising the antithrombotic effects of the drugs. Last, given the heterogeneity of immune responses in different diseases, future studies should explore the impact of antiplatelet therapies across a broader spectrum of conditions, including autoimmune diseases, chronic viral infections, and sepsis, to determine whether these drugs can be repurposed for new therapeutic applications. Although racial and ethnic differences in platelet count and reactivity have been documented in previous studies, there is a notable gap in our understanding of demographic-specific platelet-immune interactions. The current literature has limited evidence regarding how these interactions might vary across different demographic groups, largely owing to insufficient demographic stratification and small sample sizes in existing studies. Similarly, the effects of antithrombotic therapy on platelet-immune interactions across diverse populations remain poorly characterized. This knowledge gap represents an important area for future research, as understanding demographic-specific variations could lead to more personalized therapeutic approaches.

The future of antiplatelet therapies in immune regulation will depend on a more thorough understanding of their effects on inflammatory pathways and immune cell functions, as well as their long-term impact on disease progression in diverse patient populations. Until these questions are addressed, it remains premature to suggest that antiplatelet therapies can be broadly applied for immune modulation or that they represent a significant advancement in the treatment of immune-mediated diseases.

## Author Contributions

Conception and design: LM, SSF, SP, SG, MA, MM, AR, HD, SS, AD

Analysis and interpretation: LM, IC, DM, SS, AD

Data collection: LM, IC, DM, SS, AD

Writing the article: LM, IC, DM, SSF, SP, SG, MA, MM, AR, SS, AD

Critical revision of the article: LM, IC, DM, SSF, SP, SG, MA, MM, AR, HD, SS, AD

Final approval of the article: LM, IC, DM, SSF, SP, SG, MA, MM, AR, HD, SS, AD

Statistical analysis: LM, IC, DM, SSF, SP, SG, MA, MM, AR, HD, SS, AD

Obtained funding: Not applicable

Overall responsibility: AD

SS and AD contributed equally to this article and share senior authorship.

## Funding

None.

## Disclosures

None.
